# A large-scale estimate on the relationship between language and motor lateralization

**DOI:** 10.1038/s41598-020-70057-3

**Published:** 2020-08-03

**Authors:** Julian Packheiser, Judith Schmitz, Larissa Arning, Christian Beste, Onur Güntürkün, Sebastian Ocklenburg

**Affiliations:** 1 Department of Biopsychology, Institute for Cognitive Neuroscience, Ruhr University Bochum, Universitätsstraße 150, 44780 Bochum, Germany; 2 School of Medicine, University of St Andrews, St Andrews, UK; 3 Department of Human Genetics, Ruhr University Bochum, Bochum, Germany; 4 Cognitive Neurophysiology, Department of Child and Adolescent Psychiatry, Faculty of Medicine Carl Gustav Carus, TU Dresden, Dresden, Germany; 5 Department of Psychology, University of Duisburg-Essen, Essen, Germany

**Keywords:** Language, Neuroscience, Cognitive neuroscience

## Abstract

Human language is dominantly processed in the left cerebral hemisphere in most of the population. While several studies have suggested that there are higher rates of atypical right-hemispheric language lateralization in left-/mixed-handers, an accurate estimate of this association from a large sample is still missing. In this study, we comprised data from 1,554 individuals sampled in three previous studies in which language lateralization measured via dichotic listening, handedness and footedness were assessed. Overall, we found a right ear advantage indicating typical left-hemispheric language lateralization in 82.1% of the participants. While we found significantly more left-handed individuals with atypical language lateralization on the categorical level, we only detected a very weak positive correlation between dichotic listening lateralization quotients (LQs) and handedness LQs using continuous measures. Here, only 0.4% of the variance in language lateralization were explained by handedness. We complemented these analyses with Bayesian statistics and found no evidence in favor of the hypothesis that language lateralization and handedness are related. Footedness LQs were not correlated with dichotic listening LQs, but individuals with atypical language lateralization also exhibited higher rates of atypical footedness on the categorical level. We also found differences in the extent of language lateralization between males and females with males exhibiting higher dichotic listening LQs indicating more left-hemispheric language processing. Overall, these findings indicate that the direct associations between language lateralization and motor asymmetries are much weaker than previously assumed with Bayesian correlation analyses even suggesting that they do not exist at all. Furthermore, sex differences seem to be present in language lateralization when the power of the study is adequate suggesting that endocrinological processes might influence this phenotype.

## Introduction

One of the best known functional hemispheric asymmetries is the lateralization of the human language system^1^. A number of studies have demonstrated that about 85–90% of the population shows a left-hemispheric dominance in several language-related tasks^2–6^. Since speech impairment syndromes such as aphasias in patients with unilateral brain damage were found to be less severe in left-handers^7^, it was assumed that these two asymmetries are to some extent associated and may even share a common genetic foundation^8,9^.

Several studies investigated the relationship between language lateralization and handedness on the categorical level, i.e. classification of individuals into for example left- and right-handers as well as those with a left- and right-hemispheric dominance in language processing. Using both behavioral as well as neuroimaging techniques, it was found that about 90–95% of right-handers demonstrate typical left-hemispheric language lateralization^3,10^. In left-handers, the prevalence of typical language lateralization has however been found to be reduced (about 70–85%^11,12^). A meta-analysis by Carey and Johnstone^13^ investigated the relationship between handedness and language lateralization in both healthy as well as patient samples and found a 20% left hemisphere dominance increase in right-handed individuals compared to individuals with left motor preferences. Since most individuals demonstrating atypical handedness are thus nonetheless left dominant in their cerebral specialization for language processing, the link between these two phenotypes appears to be moderate at best, however. This is supported by recent findings from van der Haegen et al.^14^ who investigated language lateralization using both a dichotic listening task and silent word task during fMRI and found only a small association between handedness and language lateralization. One significant problem using categorical analyses is that there is no universal classification system. Therefore, categorical analyses can only provide limited insight into the association between motor and language lateralization as it depends on mostly arbitrary thresholds. This can however be resolved if these lateral biases are investigated with a continuous classification system using for example lateralization quotients (LQs) that represent both the direction and the strength of a lateral preference.

So far, only a handful of studies have investigated correlations between LQs of handedness and language dominance. Badzakova-Trajkov et al.^15^ for example used fMRI to measure asymmetrical frontal activation during a word generation task in 155 adult participants. They found a moderate correlation (r = 0.357) between frontal asymmetries and handedness LQs. However, only 13% of the variance in hand preference could be explained by language asymmetry. To confirm that this pattern is similar in children, Groen et al.^16^ assessed hand dominance and language processing in 57 children using a variety of handedness measures (e.g. long and short forms of the Edinburgh Handedness Inventory (EHI^17^), a peg-moving task^18^ and reaching tasks). They also found weak to moderate correlations (from r = 0.13 to r = 0.40) depending on the measure for hand preference concluding that the association between the bias in handedness and language is small- to medium-sized. Brysbaert^19^ investigated the relationship between handedness using a tachistoscopic visual half field (VHF) word naming task across two independent samples and could not identify any meaningful correlation with language lateralization (correlations were below 0.2 in both experiments). In line with this result, Somers et al.^20^ found no predictive value of handedness for language lateralization in a study investigating 310 left-handers calling into question whether there truly is a link between these phenotypes.

Besides handedness, footedness as another motor asymmetry has also been investigated for its predictive value of language lateralization. Searleman^21^ assessed language lateralization in 373 individuals and found that surprisingly footedness and not handedness was the single best predictor. Elias and Bryden^22^ complemented these results as they also found a higher predictive power of footedness compared to handedness for the language lateralization of 32 participants. A potential explanation why footedness could be a better predictor compared to handedness lies in the purity of the measure. Footedness is less likely to be influenced by cultural norms and social teaching which has been demonstrated repeatedly for handedness^23,24^. However, these findings have since never been replicated and the relationship between footedness and language lateralization remains an open question, especially given the fact that Brysbaert^19^ could not demonstrate a significant correlation between footedness and language lateralization.

A major problem of previous research investigating correlations between language lateralization and motor asymmetries pertains to the small to moderate sample sizes employed in the respective studies. If sample sizes are inadequate, it raises the likelihood to miss a present effect due to a lack of power or report spurious effects due to sampling errors. Furthermore, low sample sizes do not allow for a precise estimate of the effect size due to high variance in the data^25^. Given that motor asymmetries such as handedness and footedness and language lateralization are heavily skewed in the population^5,26^, a substantial sample size is needed to adequately represent both atypical motor as well as atypical language lateralization with sufficient power^27^. However, no study so far has investigated the correlation between these two variables on a large scale.

A suitable way to assess language lateralization in large cohorts is the dichotic listening task which generally shows a reliable right ear advantage indicating left-hemispheric language processing^28–31^. Dichotic listening tasks have been validated to accurately assess lateralized language processing using a large variety of neuroscientific methods, for example fMRI^32–34^, PET^35^ or individuals with chronic or temporary hemispheric lesions^36–39^. The right ear advantage can be found even in the absence of auditory stimuli as demonstrated via auditory imagery tasks^40–42^. Furthermore, it can be modulated through attention and unilateral high frequency stimulation via TMS^43,44^ but seems to be unaffected by constant stimulation applied through tDCS^45^. In the present study, we used dichotic listening to investigate language lateralization in a large cohort of n = 1,554 participants. The sample comprised data from three previous studies investigating the genetic basis of language lateralization and handedness^46–48^. We assessed LQs of handedness using the EHI^17^ and footedness using the Waterloo Footedness Questionnaire (WFQ)^49^ to identify the relationship with motor biases. We furthermore investigated sex differences in language lateralization, handedness and footedness as sex has been suggested to be a moderator for all these phenotypes^50–52^. The aim of the study was to identify the degree of association between motor biases such as handedness or footedness and language lateralization in a large cohort of healthy participants. Based on previous findings, we hypothesize that the extent of left-hemispheric language lateralization will be more pronounced in both right-handers and right-footers whereas left-handers and left-footers show a stronger right-hemispheric dominance in language processing.

## Methods

### Participants

No new participants were tested for this study. All data was gathered from previous publications. Overall, data from 1554 healthy participants (1,011 women) recruited in three previous studies (n = 524 from Ocklenburg et al.^46^, n = 971 from Beste et al.^48^, n = 59 from Schmitz et al.^47^) were evaluated in the present study. The mean age of the participants was 24.42 years (SD 5.70, range 18–66 years). All participants were also tested for normal hearing ability of both ears using an audiometric screening before the experiment to ensure the validity of the dichotic listening task. Participants provided written informed consent and were treated in accordance with the declaration of Helsinki. The procedures in all three studies were approved by the relevant ethics committees.

### Test procedure

The details of each individual study’s procedure can be found in the respective publication. This paragraph will therefore focus on the assessment of motor and language lateralization in all three studies.

After providing written informed consent, participants filled out demographic and motor preference questionnaires. Handedness and footedness were assessed via the EHI and the WFQ, respectively. The EHI comprises 10 items asking participants with which hand they prefer to perform a variety of everyday tasks (writing, throwing a ball, drawing, using scissors, brushing teeth, using a knife, using a spoon, using a broom, striking a match and opening a box-lid/jar). They are instructed to enter a “+” sign either in a left or right column if they prefer to perform the task with the left or right hand, respectively. If they would never use the other hand for this task unless being absolutely forced to, they are instructed to enter a “++”. If there is no preference to use either hand, the participants are instructed to enter a “+” in both the left and right preference column. For the WFQ, the participants are asked to indicate if they prefer to perform five mobility and five stability tasks (ball kicking, hopping on one leg, standing on one foot, smoothing sand, stepping onto a chair, weight-shifted relaxed standing, stepping on a shovel, grasping a marble, balancing on a rail and stepping on a bug) with the left or with the right foot/leg. The preference scoring is identical to the EHI. LQs are calculated based on the formula proposed by Oldfield^17^: LQ = [(R − L)/(R + L)] × 100. Here, R represents right-sided preferences and L represents left-sided preferences. Positive LQs therefore demonstrate a right motor preference and negative LQs a left motor preference.

Language lateralization was assessed via the dichotic listening task. In the dichotic listening task, six consonant–vowel syllable pairs (/ba/, /da/, /ga/, /ta/, /ka/, /pa/) were presented via headphones in all possible combinations with one syllable being presented on the left ear and one syllable presented on the right ear. In the study of Ocklenburg et al.^46^, four blocks of 30 trials comprising each possible dichotic syllable combination were conducted and participants had to report which syllable they heard best. If the reported syllable was a right-ear presented syllable, the trial was counted as a right-sided correct response. Conversely, if the reported syllable was a left-ear presented syllable, the trial was counted as a left-sided correct response. If the reported syllable was presented on neither the left nor right ear, the trial was counted as an error. Additionally, six trials presented homonym stimulus pairs on both ears [e.g. (/ba/) left and (/ba/) right]. Responses were given using a customized response box. Stimulus duration was on average 350 ms and the intertrial interval was 2000 ms. In the studies of Beste et al.^48^ and Schmitz et al.^47^, the iDichotic app for iOS was used to perform the dichotic listening task that has been demonstrated to show a reliable right ear advantage across large cohorts and diverse cultures^29,53^. The procedure was identical to the study by Ocklenbug et al.^46^ with the exception that only one block of trials was presented to the participants and that the intertrial interval was 4000 ms. LQs were calculated analogously to the LQs of limb preferences using the formula: LQ = [(R − L)/(R + L)] × 100. Here, R represents the number of correctly identified right ear stimuli and L the number of correctly identified left ear stimuli. A positive LQ demonstrates a right ear advantage indicating left-hemispheric language dominance while a negative LQ demonstrates a left ear advantage indicating right-hemispheric language dominance.

### Statistical analyses

A one sample t-test was performed for the dichotic listening LQs to identify whether there was a consistent ear advantage in the overall sample. Furthermore, a one-sample t-test was performed for EHI and WFQ LQs to identify lateral motor biases across all participants. For dichotic listening LQs, we assessed the reliability of the measure in the subsample of Ocklenburg et al.^46^ using a split-half correlation because an unreliable dependent variable is unlikely to be modulated by independent factors^54^. Furthermore, a variable cannot correlate more with another variable than with itself^54,55^. Thus, the reliability of the measures represents the upper limit of a correlation between the measures. To determine the association between limb preferences and language lateralization, we correlated the dichotic listening LQ with the LQs for handedness and footedness. Significance levels for the correlations were Bonferroni corrected to account for multiple comparisons. We additionally conducted Bayesian correlation matrix analysis between language lateralization and handedness as well as footedness to identify how much evidence there is in favor of the hypothesis that these phenotypes are interrelated. Bayesian correlation pair analysis was performed to identify this relationship on the single subject level.

We also divided the sample into individuals with typical (right ear advantage) and atypical (left ear advantage) hemispheric language processing. Here, participants with a dichotic listening LQ between − 10 and + 10 were excluded to remove individuals that did not exhibit a demonstrable lateralization in language dominance. By means of independent t-tests, we then compared the EHI LQs and WFQ LQs between individuals with a right and left ear advantage in the dichotic listening task.

We also classified handedness and footedness as categorical variables using four different classification systems that have been used in the literature. The classification systems were as follows:

1. Left–right: EHI/WFQ LQs < 0 were classified as left-preferent and EHI/WFQ LQs of > 0 as right-preferent^56,57^
2. Left-mixed-right 40: EHI/WFQ LQs < − 40 were classified as left-preferent, EHI/WFQ LQs of >  + 40 as right-preferent and all other LQs as mixed-preferent^58–60^
3. Left-mixed-right 60: EHI/WFQ LQs < − 60 were classified as left-preferent, EHI/WFQ LQs of >  + 60 as right-preferent and all other LQs as mixed-preferent^61,62^
4. Left-mixed-right asymmetrical: EHI/WFQ LQs < − 6 were classified as left-preferent, EHI/WFQ LQs of >  + 72 as right-preferent and all other LQs as mixed-preferent (based on latent variable analysis from Tran et al.^27^)

The prevalence of these categories for individuals with typical and atypical language lateralization were then compared using Pearson’s χ^2^ tests.

We furthermore analyzed effects of sex in EHI LQs, WFQ LQs and dichotic listening LQs via independent t-tests as differential lateralization between males and females has been demonstrated in meta-analyses^50,52,63^. Correlations between EHI and dichotic listening LQs as well as between WFQ and dichotic listening LQs were for that reason also analyzed separately for males and females. The categorical analysis that was conducted for the entire sample was repeated for males and females individually as well.

## Results

**Figure 1:**
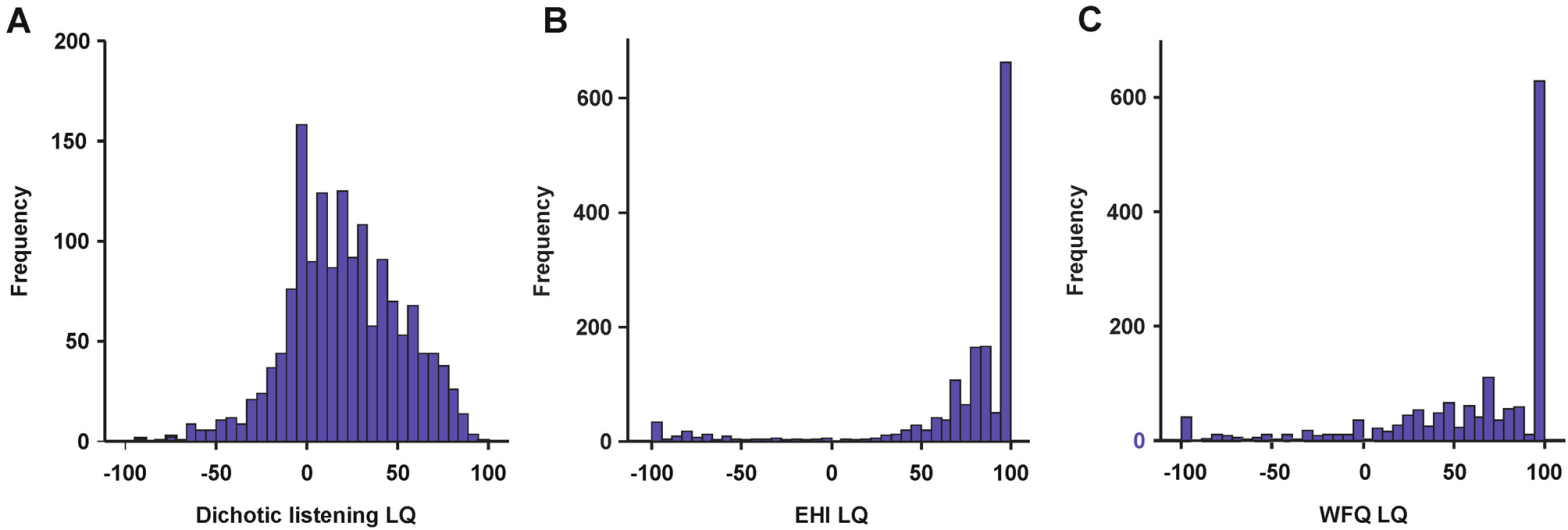
(**A**) Histogram of dichotic listening LQs. Dichotic listening LQs exhibited a unimodal distribution that was shifted to the right side indicating a right ear advantage in most individuals. (**B**) Histogram of EHI LQs. EHI LQs showed a bimodal distribution with most individuals showing a positive LQ indicating a right-hand preference. (**B**) Histogram of WFQ LQs. WFQ LQs showed a bimodal distribution with most individuals showing a positive LQ indicating a right-foot preference.

First, we aimed to identify the well-known right ear advantage in dichotic listening tasks. We found a pronounced right ear advantage in the entire sample indicating that there was a significantly stronger bias towards left-hemispheric language processing (mean dichotic listening LQ = 20.86, SD = 30.88; one sample t test: t_(1553)_ = 26.63, p < 0.001, Fig. 1A). For both EHI and WFQ LQs, there was a strong right-sided preference across all participants (Fig. 1B,C, respectively).

Reliability of dichotic listening LQs in the sample of Ocklenburg et al.^46^ was at r_(523)_ = 0.83 (p < 0.001, split-half correlation). Bless et al.^53^ had previously estimated the test–retest reliability of the iDichotic app to be at r = 0.78. Thus, a similar reliability can be assumed for our two datasets using this app.

**Figure 2:**
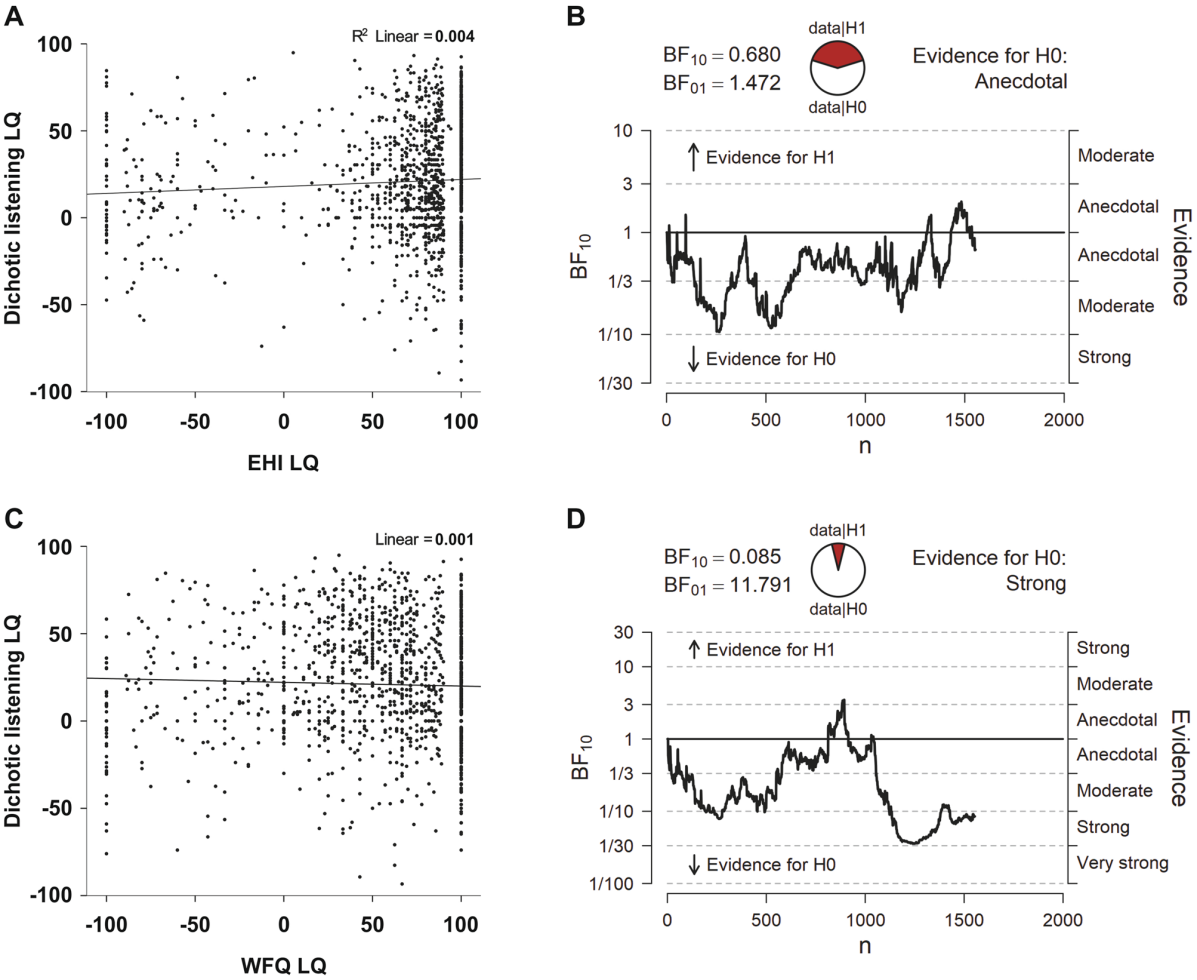
(**A**) Correlation analysis between EHI LQs and dichotic listening LQs resulted in a weak positive association. (**B**) Bayesian correlation pair analysis for the association between EHI LQs and dichotic listening LQs across the entire sample. In the top left, the BF10 and BF01 values are presented for which a value of > 1 indicates evidence in favor of the alternative (H1) or null hypothesis (H0), respectively. In the top center, the fraction of data supporting the H1 and H0 is shown. The graph below indicates how evidence in favor of the H1 and H0 develops for each individual participant in the sample. Overall, there was anecdotal evidence in favor of the H0. (**C**) Correlation analysis between WFQ LQs and dichotic listening LQs did not show any association. (**D**) Same as in B, but for the association between WFQ and dichotic listening LQs. There was overall strong evidence in favor of the H0.

Correlating dichotic listening LQs with the EHI LQ yielded a significant positive association (r_(1553)_ = 0.06, p = 0.026, Fig. 2a) indicating that language lateralization and handedness were interrelated, albeit weakly. Linear regression analysis furthermore revealed that the EHI LQ explained only 0.4% of the variance of the dichotic listening LQ. Using Bayesian correlation matrix analyses, we found anecdotal evidence in favor of the null hypothesis (BF_10_ = 0.68) indicating that there is no relationship between dichotic listening and EHI LQs. Bayesian correlation pair analysis revealed that more than half of the participants generated data in favor of the null hypothesis (Fig. 2B). For WFQ LQs, there was no significant correlation with dichotic listening LQs (r_(1553)_ = − 0.04, p = 0.322, Fig. 2C) that was also supported by strong evidence in favor of the null hypothesis using Bayesian correlation matrix analysis (BF_10_ = 0.09). Bayesian correlation pair analysis demonstrated that the large majority of the sampled individuals generated data in favor of the null hypothesis (Fig. 2D).

**Figure 3:**
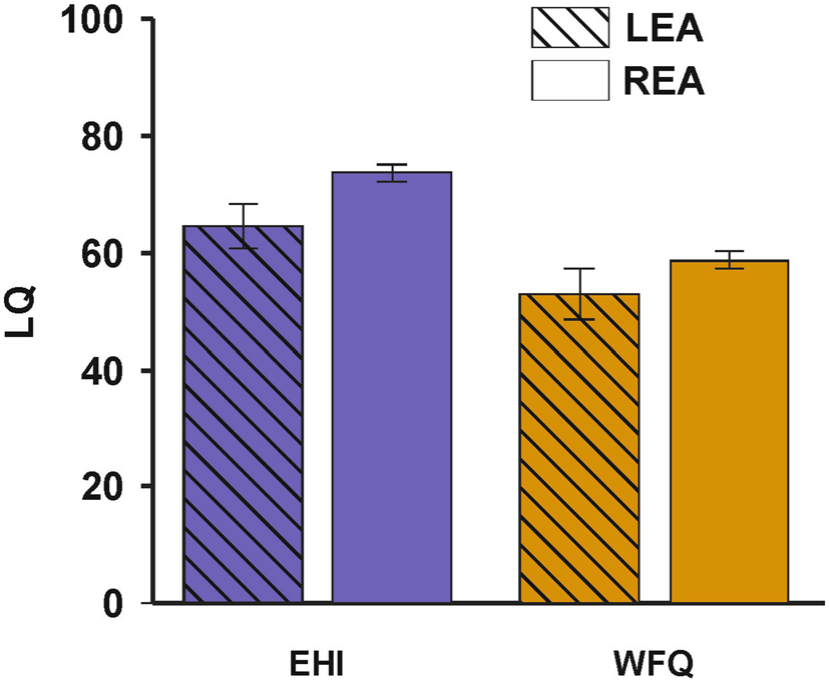
LQs for the EHI and WFQ divided for individuals showing a right ear advantage (REA) indicating typical language lateralization or a left ear advantage (LEA) indicating atypical language lateralization. Error bars represent SEM.

In a next step, we divided the cohort into individuals with typical (dichotic listening LQ > 10, n = 969) and atypical language lateralization (dichotic listening LQ < − 10, n = 211). Comparing the EHI LQs between these subgroups revealed a significant difference where individuals with typical language lateralization demonstrated a higher EHI LQ (mean LQ = 73.68, SD = 45.50) compared to individuals with atypical language lateralization (mean LQ = 64.59, SD = 55.58, t_(1178)_ = 2.52, p = 0.012, Fig. 3). There was no difference in the WFQ LQs (t_(1178)_ = 1.51, p = 0.130, Fig. 3).

We then used handedness and footedness as categorical variables using four different classification schemes.

For the left–right classification, we found that 12.3% of the participants with a left ear advantage were left-handed whereas only 7.9% of the participants with a right ear advantage were left-handed (χ^2^_(1)_ = 4.17, p = 0.041). For footedness, a similar result was found as 19.0% of the participants with a left ear advantage were left-footed whereas only 12.3% of the participants with a right ear advantage were left-footed (χ^2^_(1)_ = 6.63, p = 0.010).

For the left-mixed-right 40 classification, we found no significant difference in handedness between individuals with typical and atypical language lateralization. For footedness, there was however a significantly higher fraction of participants with left preferences in individuals with atypical (12.8%) compared to typical language lateralization (5.7%, χ^2^_(2)_ = 14.24, p = 0.001).

For the left-mixed-right 60 classification, we found that 10.4% of the participants with a left ear advantage were left-handed whereas only 5.8% of the participants with a right ear advantage were left-handed (χ^2^_(2)_ = 7.64, p = 0.022). For footedness, participants with a left ear advantage were more often left-footed compared to individuals with a right ear advantage (9.0% vs. 4.0%), but also less often mixed-footed (27.49% vs. 35.91%, χ^2^_(2)_ = 12.66, p = 0.002).

For the left-mixed-right asymmetrical classification, we found no significant differences in the prevalence of left-, mixed- or right-handedness between individuals with typical and atypical language lateralization (χ^2^_(2)_ = 5.03, p = 0.081). For footedness, participants with a left ear advantage were more often left-footed compared to individuals with a right ear advantage (17.5% vs. 10.2%), but also less often mixed-footed (29.86% vs. 43.03%, χ^2^_(2)_ = 16.69, p < 0.001).

**Figure 4:**
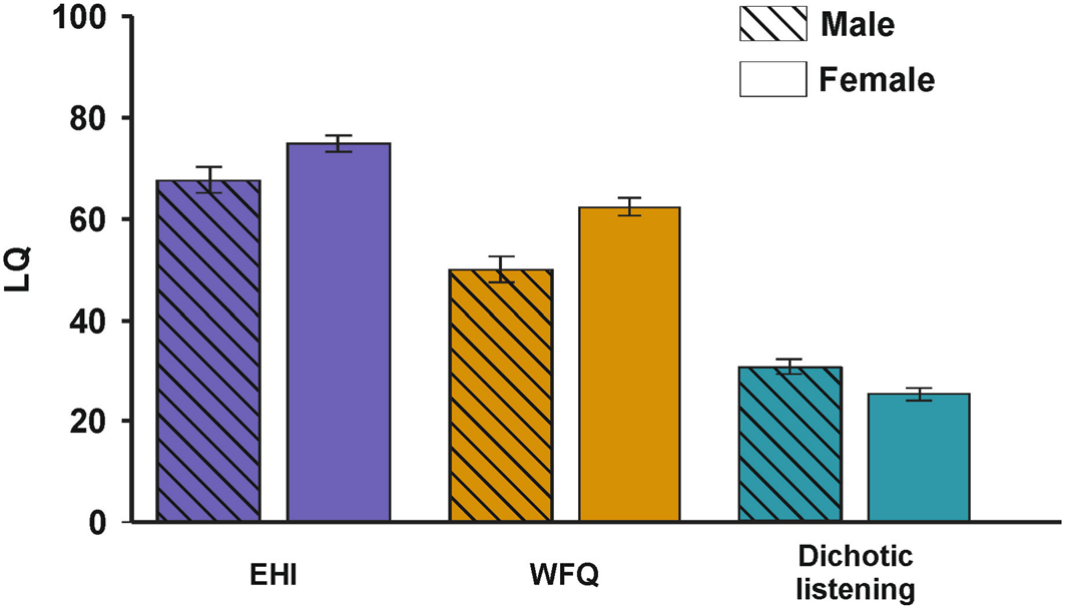
LQs for the EHI, WFQ and dichotic listening divided by sex of the participant. Error bars represent SEM.

To identify sex differences in handedness, footedness and language lateralization, we compared the EHI, WFQ and dichotic listening LQs between males and females. We found no difference in the EHI LQ (independent sample t-test: t_(1553)_ = 1.75, p = 0.079, Fig. 4, left), but a significantly lower WFQ LQ in males (t_(1553)_ = 3.58, p < 0.001, Fig. 4, center). For dichotic listening LQs, there was a significantly higher right ear advantage in males compared to females indicating stronger left-hemispheric language lateralization (t_(1553)_ = 3.29, p = 0.001, Fig. 4, right). Both for males and females, there was a weak positive correlation between the dichotic listening and the EHI LQ, but only the correlation in females reached significance (females: r_(1010)_ = 0.08, p = 0.026; males: r_(543)_ = 0.05, p = 0.265). For WFQ LQs, there was no association with dichotic listening LQ in either sex (females: r_(1010)_ = − 0.05, p = 0.109; males: r_(543)_ = 0.01, p = 0.844).

Sex differences between males and females were also investigated on the categorical level. For language lateralization, males were more often left-hemispheric dominant in their language processing compared to females (85.28% in males vs. 80.31% in females, χ^2^_(1)_ = 4.57, p = 0.032).

For the left–right classification, we found no significant difference in handedness between males and females. More males were left-footed compared to females however (16.57% in males vs. 11.86% in females, χ^2^_(1)_ = 6.69, p = 0.010).

For the left-mixed-right 40 classification, more males were mixed-handed compared to females (8.8% in males vs. 4.3% in females, χ^2^_(2)_ = 7.48, p = 0.024). For footedness, we also found increased rates of mixed-footedness in males (24.13% vs. 17.31%, χ^2^_(2)_ = 13.48, p = 0.001).

For the left-mixed-right 60 classification, there was no significant difference in handedness between males and females. For footedness, there were increased rates of mixed-footedness in males (37.38% vs. 28.78%, χ^2^_(2)_ = 13.54, p = 0.001).

For the left-mixed-right asymmetrical classification, there was no significant difference in handedness between males and females. For footedness, there were increased rates of mixed-footedness in males (42.72% vs. 35.01%, χ^2^_(2)_ = 16.03, p < 0.001).

## Discussion

In this study, we investigated a large cohort of healthy participants sampled from three previous studies to illuminate the relationship between language lateralization assessed via dichotic listening and handedness as well as footedness. We found a very weak correlation of language dichotic listening LQs with handedness LQs indicating that individuals with typical left-hemispheric language lateralization are also marginally more likely to be right-handed. Handedness LQs were also significantly reduced in individuals with atypical language lateralization. There was no significant association between dichotic listening LQs and footedness LQs.

Overall, our results are only barely in line with the notion that there is a link between language lateralization and handedness. We only found a small association with a marginal 0.4% explained variance of handedness in language lateralization. Furthermore, Bayesian correlation matrix analyses indicated that there was even anecdotal evidence in favour of the null hypothesis i.e. that there is no association between these two phenotypes. Given that our study was well-powered, it calls into question whether the moderate associations found in previous studies investigating this relationship overestimated the link between language lateralization and handedness on the continuous level^15,16^. Our results thus strongly support the findings from Brysbaert^19^ and van der Haegen et al.^14^ who could detect no or at best a small association between handedness and language lateralization. Interestingly, the former study found a significant association of language lateralization and ear preference. Since our datasets did not contain information on the participants’ ear preference, it remains to be seen if there is a stronger relationship between these variables.

The notion that the association between handedness and language dominance might have been overestimated is supported by findings on the modifiability of language lateralization due to the phase of the hormonal cycle as women show reduced language lateralization during their midluteal phase^64^. Handedness on the other hand appears to be a stable characteristic independent of endocrinological changes as no study has ever reported effects of for example sex hormones or acute stress on handedness. Further indications in line with this conclusion come from studies investigating a common genetic foundation of both phenotypes. While many genes have been identified to be associated both with handedness and language lateralization individually, none of these genes has ever been implicated to play a role in the ontogenesis of both^9^. Also, when investigating gene ontogeny (GO) sets where genes are clustered into functional groups, almost no overlap between GO sets involved in language lateralization and handedness has been identified indicating that the underlying biological cascades giving rise to the phenotypes are fundamentally different ^65^. It has to be noted however that there has been no large scale genome-wide association study (GWAS) investigating the genetic basis of language lateralization as has been done for handedness^66–69^. The handedness GWAS by Wiberg et al.^66^ performed in the UK Biobank data, was however complemented with a neuroimaging analysis in a subsample of 721 left-handers and 6,685 right-handers. The authors reported stronger functional connectivity between left- and right-hemispheric language areas (encompassing Broca’s and temporoparietal junction areas) in left-handers compared to right-handers. Interestingly, one common single nucleotide polymorphism (rs199512) was associated with handedness as well as white matter integrity of these white matter tracts. Thus, genes involved in both phenotypes might still be identified in the future that have not yet been in the scope of candidate gene or GO set studies.

In our study, we also investigated the relationship between language lateralization and footedness as previous research has suggested that footedness might be a better predictor compared to handedness for cerebral lateralization such as language dominance^22^ and emotional processing^49^. At least concerning language lateralization, our study suggests no indication that footedness explains any variance in the hemispheric dominance for language processing on the continuous level in line with the results of Brysbaert^19^ who could not find a significant association between language lateralization and footedness. There was strong evidence using Bayesian correlation matrix analysis that the null hypothesis applies indicating that footedness is not directly related to language lateralization. However, in the categorical analyses regardless of classification, we found higher rates of left- or mixed-footedness in individuals with atypical language lateralization. This result indicates that there is a link between atypical footedness and atypical language lateralization, but the strength of this atypicality is uncorrelated (e.g. an individual might be strongly left-footed, but only marginally right-lateralized in language processing and vice versa). Given that this result was consistent across classification schemes, footedness could play a role in predicting atypical brain lateralization in general, but not in predicting its strength.

Regarding sex differences between males and females, we first investigated the known findings of increased atypical handedness and footedness in the male population^50,52^. Albeit the effects were strong for footedness in this study, there was only a trend towards a reduced EHI LQ in males. Furthermore, only in one classification system (left-mixed-right 40) could we find increased rates of atypical handedness in males. A possible reason for this finding could be that per 100 individuals, there are only 2.1 more left-handed males than females^50^ indicating that the overall effect is rather small and hard to detect even in larger samples.

We found that males exhibited a stronger right ear advantage indicating stronger language lateralization to the left hemisphere. A few studies have indicated that women have a more bilateral language processing compared to men (e.g. Shaywitz et al.^70^) which has been hypothesized to be advantageous in verbal processing^71^. Hausmann and Güntürkün^64^ proposed that the more bilateral processing of lexical tasks in women is mediated by the sex hormone progesterone that influences information transfer across the corpus callosum. However, two meta-analyses on influences of sex in language lateralization could not determine a sex difference as there was no stronger right ear advantage in dichotic listening tasks in males compared to females, nor a stronger left-hemispheric activation during lexical processing in functional imaging data^72,73^. Since we found a rather strong effect in the dichotic listening LQs, it however raises the question whether these meta-analytical findings are conclusive. Lust et al.^74^ for example found that the level of language lateralization in children depends on prenatal testosterone levels, therefore being naturally increased in boys compared to girls. More research on sex differences in language lateralization is thus needed as language lateralization seems to depend, at least in part, on endocrinological processes^64,74^.

The present study, while providing a well-powered sample on the relation between language and motor lateralization, is not without limitations. One important limitation pertains to the fact that language lateralization was assessed via dichotic listening rather than functional imaging. Since dichotic listening mismatches about 5–8% of the tested individuals in right-handers with regards to language lateralization^36^, a small subset of participants might have been misclassified adding noise to the data. Thus, the observed correlations could be slightly higher or lower. Due to the large sample, we however believe that the presented effects provide an accurate estimate despite this shortcoming. Furthermore, handedness and footedness were exclusively measured via self-report questionnaires. The participants therefore did not have the opportunity to perform the task to identify which hand or foot they prefer. This is more problematic for footedness as some items are not everyday activities calling into question whether all participants were certain which foot they favored for a certain task. It has to be noted however that both the EHI and the WFQ match very well with neurophysiological differences between left- and right-handers and left- and right-footers^59^ providing some validity also on the neural level to these questionnaires.

In conclusion, we could only find minor associations between handedness and no association between footedness and language lateralization on the continuous level. Bayesian correlation analyses suggested that there is no relation between handedness or footedness and language lateralization calling into question whether motor asymmetries and hemispheric dominance in language processing are interrelated and whether they share a common genetic basis.

### Statement for the approval of the procedures

All experimental procedures in this study were approved by the local ethics committee of the psychology faculty at the Ruhr University Bochum. All participants were treated in accordance with the declaration of Helsinki.

## Data Availability

All data will be made available upon request by the authors.
