# Author Correction: A large-scale estimate on the relationship between language and motor lateralization

**DOI:** 10.1038/s41598-020-75423-9

**Published:** 2020-11-12

**Authors:** Julian Packheiser, Judith Schmitz, Larissa Arning, Christian Beste, Onur Güntürkün, Sebastian Ocklenburg

**Affiliations:** 1 Department of Biopsychology, Institute for Cognitive Neuroscience, Ruhr University Bochum, Universitätsstraße 150, 44780 Bochum, Germany; 2 School of Medicine, University of St Andrews, St Andrews, UK; 3 Department of Human Genetics, Ruhr University Bochum, Bochum, Germany; 4 Cognitive Neurophysiology, Department of Child and Adolescent Psychiatry, Faculty of Medicine Carl Gustav Carus, TU Dresden, Dresden, Germany; 5 Department of Psychology, University of Duisburg-Essen, Essen, Germany

Correction to: *Scientific Report * 10.1038/s41598-020-70057-3, published online 03 August 2020

The Acknowledgements section in this Article is incomplete.

“This study was supported by the Deutsche Forschungsgemeinschaft through the Research Training Group “Situated Cognition” (GRK 2185/1). We acknowledge support by the DFG Open Access Funds of the Ruhr-Universität Bochum.”

should read:

“This study was supported by grants of the Deutsche Forschungsgemeinschaft (GU227/16-1; BE4045/26-1) as well as through the DFG Research Training Group “Situated Cognition” (GRK 2185/1). This study was also supported by the Ruhr University Bochum Cutting-Edge grant. We acknowledge support by the DFG Open Access Funds of the Ruhr-Universität Bochum.”

